# Analysis of the frequency and spectrum of mutations recognised to cause familial hypercholesterolaemia in routine clinical practice in a UK specialist hospital lipid clinic^☆^

**DOI:** 10.1016/j.atherosclerosis.2013.04.011

**Published:** 2013-07

**Authors:** Marta Futema, Ros A. Whittall, Amy Kiley, Louisa K. Steel, Jackie A. Cooper, Ebele Badmus, Sarah E. Leigh, Fredrik Karpe, H. Andrew W. Neil, Simon Broome Register Group, Steve E. Humphries

**Affiliations:** a Centre for Cardiovascular Genetics, British Heart Foundation Laboratories, Institute of Cardiovascular Science, Rayne Building University College London, London WC1E 6JF, UK; b OCDEM, Radcliffe Department of Medicine, University of Oxford, Churchill Hospital, Oxford OX3 7LE, UK; c NIHR School of Primary Care Research, Department of Primary Care Health Sciences, University of Oxford, Oxford, UK

**Keywords:** Cholesterol, Diagnostics, Familial hypercholesterolaemia, Genetic, Lipids, Mutations, FH, familial hypercholesterolaemia, ARMS, amplification refractory mutation system, HRM, high resolution melting, MLPA, multiplex ligation probe-dependent amplification, TC, total cholesterol, TG, triglycerides, CHD, coronary heart disease, DLCN, Dutch Lipid Clinic Network, NGS, next generation sequencing, NICE, National Institute for Health and Clinical Excellence, DFH, definite FH, PFH, possible FH, UH, unclassified hypercholesterolaemia

## Abstract

**Aim:**

To determine the frequency and spectrum of mutations causing Familial Hypercholesterolaemia (FH) in patients attending a single UK specialist hospital lipid clinic in Oxford and to identify characteristics contributing to a high mutation detection rate.

**Methods:**

289 patients (272 probands) were screened sequentially over a 2-year period for mutations in *LDLR*, *APOB* and *PCSK9* using standard molecular genetic techniques. The Simon Broome (SB) clinical diagnostic criteria were used to classify patients and a separate cohort of 409 FH patients was used for replication.

**Results:**

An FH-causing mutation was found in 101 unrelated patients (*LDLR* = 54 different mutations, *APOB* p.(Arg3527Gln) = 10, *PCSK9* p.(Asp374Tyr) = 0). In the 60 SB Definite FH patients the mutation detection rate was 73% while in the 142 with Possible FH the rate was significantly lower (27%, *p* < 0.0001), but similar (14%, *p* = 0.06) to the 70 in whom there was insufficient data to make a clinical diagnosis. The mutation detection rate varied significantly (*p* = 9.83 × 10^−5^) by untreated total cholesterol (TC) levels (25% in those <8.1 mmol/l and 74% in those >10.0 mmol/l), and by triglyceride levels (20% in those >2.16 mmol/l and 60% in those <1.0 mmol/l (*p* = 0.0005)), with both effects confirmed in the replication sample (*p* for trend = 0.0001 and *p* = 1.8 × 10^−6^ respectively). There was no difference in the specificity or sensitivity of the SB criteria versus the Dutch Lipid Clinic Network score in identifying mutation carriers (A_ROC_ respectively 0.73 and 0.72, *p* = 0.68).

**Conclusions:**

In this genetically heterogeneous cohort of FH patients the mutation detection rate was significantly dependent on pre-treatment TC and triglyceride levels.

## Highlights

• 54 different *LDLR* mutations found in a cohort of 272 FH probands.
• The cohort was found to be genetically heterogeneous with no specific FH mutation.
• Mutation detection rate was highly dependent on pre-treatment TC and TG levels.
• No difference in specificity/sensitivity between 2 clinical FH diagnosis approaches.
• Inadequate LDL-C reduction marks the need for more effective lipid-lowering therapy.

## 1 Introduction^1^

Familial Hypercholesterolaemia (FH) is a common autosomal dominant disease caused by mutations affecting the plasma clearance of LDL-cholesterol (LDL-C) [1]. FH patients have elevated levels of total cholesterol (TC) and LDL-C from birth, and if untreated, develop coronary heart disease (CHD) by the age of 55 in 50% of men and 30% of women [2]. The clinical phenotype of FH is known to be due to mutations in three genes encoding proteins involved in the uptake of LDL-C from the plasma, *LDLR*, *APOB* and *PCSK9*. In the UK, the Simon Broome Register criteria are used for the clinical diagnosis of FH, whereas other European countries may use a score developed by the Dutch Lipid Clinic Network (DLCN) [1,3]. The estimated frequency of heterozygous FH in the UK is 1 in 500 to 1 in 600 [4], and about 120,000 individuals would therefore be predicted to be affected by FH, although only about 15% of them are currently being treated at lipid clinics [5].

To date, there are over 1200 different *LDLR* mutations reported [6] but only one common *APOB* (c.10580G > A, p.(Arg3527Gln)) and one *PCSK9* (c.1120G > T, p.(Asp374Tyr)) [7]. The spectrum of FH mutations in Europe varies between countries, from Greece with only six mutations, which account for 60% of FH in the country, to the Netherlands with one of the most heterogeneous spectrum [8,9]. In the UK there are over 200 different mutations reported [10], which is similar to other western countries. *LDLR* mutations include mainly single nucleotide changes, which alter the amino acid composition of the mature protein, affect the correct splicing of the transcript, or binding of key transcription factors, if located in the promoter region (publication in revision, Khamis A et al.). Large deletions and insertions account for approximately 5–6% of all FH genetic defects [10,11]. The high number of different FH mutations makes genetic testing labour-intensive and costly, which has encouraged the development of novel assays and techniques such as next-generation sequencing (NGS) for diseases like FH [12].

Statin drug therapy significantly reduces the morbidity and mortality from premature coronary disease in FH, particularly if affected individuals are identified and treated in childhood or early adulthood [13–15]. The UK National Institute for Health and Clinical Excellence (NICE) guidelines published in 2008 recommended that all FH patients be offered a DNA test to confirm the diagnosis and that identified mutations should be used as the basis for cascade testing of first-degree relatives of index cases. Patients newly identified by such screening can then be offered treatment to reduce the risk of premature cardiac events [16]. DNA testing for FH has also been shown to complement cholesterol measurement in the management of affected individuals [17].

This study is aimed to assess the frequency and spectrum of mutations recognised to cause FH among patients attending the Oxford Lipid Clinic. The frequency of specific mutations in the UK differs between areas, with p.(Glu101Lys) being the most common in Manchester [18], p.(Arg350*) in South of England [19], and p.(Cys184Tyr) in Glasgow [20]. This study examined whether there are any specific mutations that occur with an unexpected frequency among patient attending the Oxford lipid clinic, which is a specialist clinic with a catchment population of over 620,000 people [4]. The correlation between the measured pre-treated cholesterol, pre-treated triglycerides and the mutation detection rate was also assessed to test the hypothesis that the individuals carrying a FH mutation have higher pre-treatment cholesterol levels and lower triglyceride level compared to those with no mutation. The likelihood of identifying mutation carriers was compared using two different clinical diagnostic criteria: the Simon Broome criteria and the DLCN score. In addition, the study examined whether the effectiveness of lipid-lowering therapy varied between patients with different genetic causes of FH.

## 2 Materials and methods

### 2.1 Patient selection criteria

The Oxford FH cohort comprised individuals who attended sequentially the Oxford Lipid Clinic, in England over the period 2009–2011. All participants were Caucasian, aged 18 or over, and were diagnosed with either definite FH (DFH) or possible FH (PFH) using the Simon Broome clinical diagnostic criteria [3,21], or as having unclassified hypercholesterolaemia (UH) which was defined as a total cholesterol and/or LDL-C concentration above the Simon Broome criteria cut off (respectively >7.5 mmol/l and/or >4.9 mmol/l) but with no family history of early CHD or with no such family history that could be elicited. The Simon Broome diagnostic criteria for FH exclude subjects with a triglyceride level of >4.5 mmol/l and none of the patients exceeded this level. There were a total of 289 patients in the cohort, of which 272 probands were apparently unrelated. The Simon Broome British Heart Foundation study (SBBHF) of 409 individuals was used for the replication of the FH clinical diagnosis methods comparison between the Simon Broome FH criteria and the DLCN score, and for the testing of the mutation detection association with TC and TG quartiles. This was a cross-sectional comparison of white patients aged 18 years or more with treated DFH with and without clinically documented CHD recruited from clinics in London, Oxford and Manchester. Recruitment methods, inclusion and exclusion and diagnostic criteria have been described previously [21]. The cohort consisted of 328 FH-mutation positive (FH/M+) and 81 FH-mutation negative (FH/M-) patients and the baseline characteristics of the cohort are shown in Supplemental Data Table 1 (pre-treatment TG levels were not available for the analysis).

### 2.2 Molecular genetic analysis

Genomic DNA was isolated from whole blood using standard methods [22]. Samples were first screened for the 20 most common UK mutations, including p.(Arg3527Gln) in *APOB* and p.(Asp374Tyr) in *PCSK9*, with a commercially available Elucigene™ FH20 (Gen-Probe Life Sciences, UK) Amplification Refractory Mutation System (ARMS) kit [11]. Next the promoter, intron–exon junctions and the coding sequence of the *LDLR* gene (NM_000527.2) were screened by High Resolution Melting (HRM) method using the Rotor-Gene 6000 [23]. The *LDLR* gene was then screened for gross deletions and insertions using Multiplex Ligation-dependent Probe Amplification (MLPA) assay, SALSA P062 from MRC-Holland (Amsterdam), on the 96-capilary ABI 3730 XL and GeneMarker software. Mutations were designated according to the Human Genome Variation Society guidelines (http://www.hgvs.org/mutnomen/).

### 2.3 Mutation prediction

Novel *LDLR* variants were assessed by *in silico* mutation prediction tools, including PolyPhen2, SIFT, and Mutation Taster. Analysis of conservation and structure, as previously described [6], were additionally used for variants with an ambiguous effect. Mutation nucleotide numbers were designated using the *LDLR* sequence reported (https://grenada.lumc.nl/LOVD2/UCL-Heart/home.php?select_db=LDLR) with the cDNA numbering beginning with A (A = 1) of the initiating ATG codon.

### 2.4 Statistical analysis

All statistical analyses were carried out using R (R Foundation for Statistical Computing, Vienna, Austria, ISBN 3-900051-07-0). Matched pre- and post-treatment LDL-C values were available for 104 patients (69 mutation negative, 35 mutation positive). Concentrations of serum cholesterol, LDL-C, HDL-C and triglyceride were not normally distributed, and were presented as geometric means with an approximate standard deviation. Matched pre-treatment TC and TG values were available for 159 patients (62 mutation positive). Dutch scores were calculated using the weights for diagnostic traits as described [1]. SBBHF study subjects were scored zero for arcus cornealis since there was no information available for this item. SBBHF LDL-C measures were post-treatment and we therefore estimated pre-treatment values for use in the score assuming a reduction of 40% in LDL-C with treatment. The ability of the criteria to discriminate between mutation carriers and non-carriers was assessed by the area under the ROC curve using combined data from both studies. Areas were compared using the method described by Delong [24]. Dutch scores were adjusted for study differences before construction of the ROC curves. A *p* value of <0.05 was taken as significant.

## 3 Results

### 3.1 Patient characteristics

In total, 289 (272 probands) FH patients were screened for FH mutations in three genes, *LDLR*, *APOB* and *PCSK9*. Characteristics of the patients recruited for the study was shown in Table 1. The majority (52%) of individuals had the clinical diagnosis of PFH with 23% being DFH. 26% of the patients could not be classified (UH) due to a lack of family history of early CHD or the patient was unaware of the family history. There was no significant difference in age or in the male/female ratio between the groups. The mean pre-treatment cholesterol differed significantly between groups (*p* < 0.0001), with DFH having the highest TC (9.79 mmol/l) and LDL-C (6.93 mmol/l) levels. PFH and UH groups had similar pre-treatment TC and LDL-C levels. The highest pre-treatment TG levels (2 mmol/l) was observed in the UH patients, and it was significantly different between the groups (*p* = 0.004), however similar to PFH (*p* = 0.162).

### 3.2 Mutation spectrum

A FH-causing variant was found in 101 individuals, of which the most frequently observed was *APOB* p.(Arg3527Gln), present in 11 individuals (10 probands). There were 54 different *LDLR* mutations, which were found in 90 patients and accounted for 89% of all observed mutations. The most commonly observed mutations in *LDLR* were c.301G > A (p.(Glu101Lys)) present in six probands, c.259T > G (p.(Trp87Gly)), c.313+1G > A, c.680_681delAC (p.(Asp227Glyfs*12)), c.681C > G (p.(Asp227Glu)), c.1116_1119dupGGGT (p.(Glu374fs*8)), and c.2054C > T (p.(Pro685Leu)), all observed in three FH probands. Most of the changes occurred in exons 4 and 10, which are the longest *LDLR* exons. There were no FH-causing variants found in exons 1, 12, 16 and 18 of the *LDLR*. The MLPA analysis of *LDLR* detected large gene rearrangements in 11 probands, which accounted for over 10% of all Oxford FH mutations. All observed mutations were summarised in Supplemental Data Table 2. There were no patients with the *PCSK9* p.(Asp374Tyr) mutation in this cohort.

### 3.3 Novel *LDLR* variants

There were 12 novel *LDLR* variants, which were not reported on the UCL FH database (Table 2). These included one promoter variant (c.-121T > C), which was further studied and proved to affect a transcription factor binding site and reduced luciferase activity by 50 ± 8% suggesting strongly that this variant is FH-causing (publication in revision, Khamis A et al.). Six non-synonymous changes were identified of which two were located in exon 4 of the gene, one nonsense mutation (c.898A > T, p.(Arg300*)), three small rearrangements, of which two lead to a frame shift and premature termination, and one large gene rearrangement – duplication of exon 11, predicted to cause frame shift and premature termination. Using *in silico* prediction tools, all novel variants were found to be pathogenic. Deletion of nine amino acids in c.667_693del (p.(Lys223_Cys231del)) was assessed using conservation and structure analysis [6]. This region includes the highly conserved D-x-S-D-E motif (residues 224–228) in exon 4. The secondary structure of this region and the coordination of a calcium cation are crucial for pH dependent recycling of the LDL-R peptide. Therefore, deletion of residues 227 and 228, which are directly involved in calcium coordination and removal of the disulphide bridge formed between residues 231 and 216, is very likely to have a pathogenic effect (Supplemental Data Fig. S1). All novel mutations were submitted to the UCL FH database [6].

### 3.4 Mutation detection rate and patients' lipid levels

**Fig. 1:**
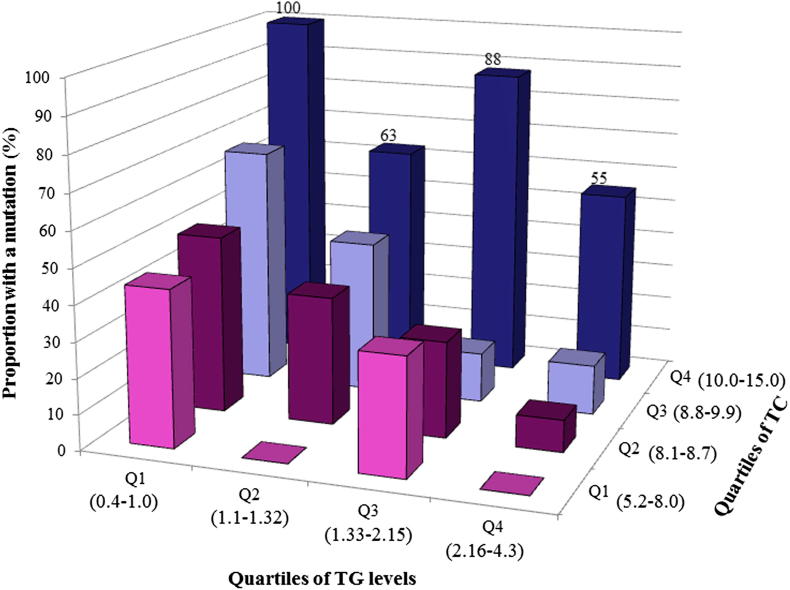
The FH mutation detection rates by combined pre-treatment TC quartiles and pre-treatment TG. The range of each quartile is shown in brackets (mmol/l).

An FH-causing mutation was detected in 73% DFH and in 27% PFH patients (probands), with 10 mutations found in the unclassified hypercholesterolaemia group (14% of probands) and overall this difference in detection rate was highly statistically significant (*p* < 0.0001). The mutation detection rate was significantly different by pre-treatment TC levels quartile (*p* = 9.83 × 10^−5^), and in the top quartile (pre-treated TC of 10.0–15.0 mmol/l) an FH mutation was found in 74% of patients (Table 3). The mutation detection rate also differed significantly depending on pre-treatment TG levels (*p* = 0.0005) (Table 3). Individuals with the lowest TG levels (0.4–1.0 mmol/l) had the highest detection rate (60%), which decreased to 20% for patients in the top quartile (2.16–4.3 mmol/l). When combined, the mutation detection rate was 100% in individuals whose TG levels were in the lowest quartile and TC levels in the top quartile compared to a less than 5% detection rate in those with TG levels in the highest quartile and TC levels in the lowest quartile (Fig. 1). These findings were replicated in the SBBHF cohort, where the highest mutation detection rate of 90% was observed in patients with pre-treatment TC above 11.6 mmol/l, which decreased to 68% in those with TC equal to or below 8.7 mmol/l, confirming the association of pre-treatment TC levels with the mutation detection rate (*p* = 0.0001). Because pre-treatment TG levels were not available, the post-treatment TG levels were used and this also showed high association (*p* = 1.8 × 10^−6^) (Table 3).

We also compared the specificity and sensitivity of correctly identifying mutation carriers by using the Simon Broome FH criteria compared to the Dutch Lipid Clinic Network (DLCN) score, 220 Oxford FH patients had data available to allow the DLCN Score to be calculated. As shown in Table 4 the mutation detection rate significantly increased (*p* = 0.004) with rising DLCN Score. The number of DFH individuals diagnosed using the Simon Broome criteria was significantly correlated with the DLCN score (2.6 × 10^−7^), indicating high similarity of both methods. When we compared the discriminatory power of the two methods using the A_ROC_ statistic, both methods performed well (ROC values for SB = 0.73 (95% CI = 0.68–0.77) vs. DLCN = 0.72 (95% CI = 0.69–0.77), *p* values for difference = 0.68) (see Supplemental Data Fig. 2), confirming that the sensitivity and specificity of both clinical diagnostic approaches were not different.

### 3.5 Mutation carriage and lipid levels pre- and post-treatment

**Fig. 2:**
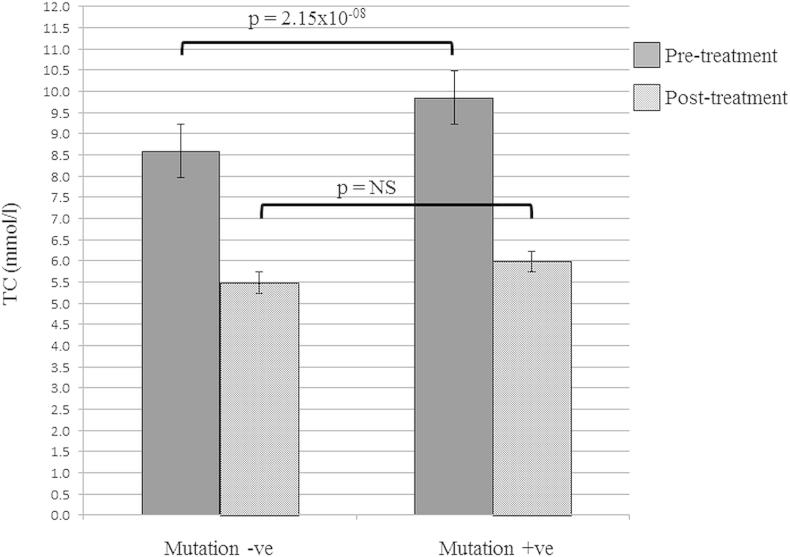
Mean pre-treatment and post-treatment TC levels in mutation negative (−ve) vs. mutation positive (+ve) patients. *P* values shown from Welch Two Sample *t*-test. NS = not significant.

**Fig. 3:**
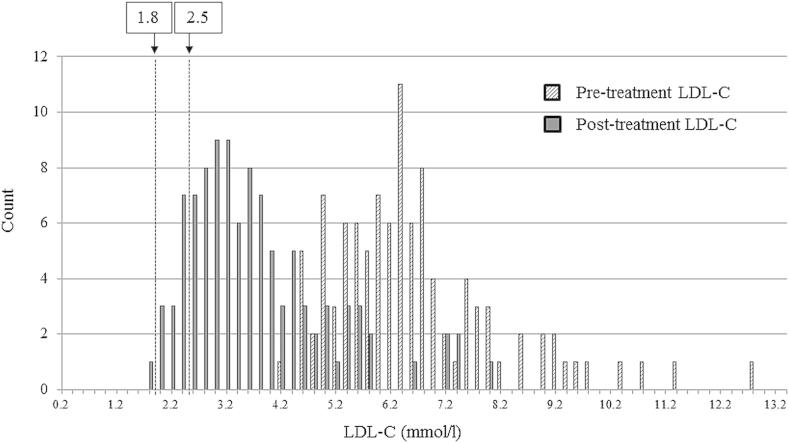
LDL-C levels distribution before and after treatment. Dashed lines at 1.8 mmol/l and 2.5 mmol/l indicate the recommended post-treatment levels for FH individuals with CVD and with high risk of CVD, respectively [25].

The mean level of pre-treatment TC in patients with any detected FH-causing mutation was significantly higher than in those with no mutation (*p* = 2.15 × 10^−08^, Welch Two Sample *t*-test) (Fig. 2). As shown in Supplemental Data Fig. 2, the highest pre-treatment TC was seen in the patients with any *LDLR* mutation (9.81 mmol/l, SD = 1.52), followed by the familial defective ApoB (FDB) patients (9.12 mmol/l, SD = 0.85), while the mutation-negative patients had the lowest pre-treatment TC (8.47 mmol/l, SD = 1.36). The difference between the TC levels in *LDLR* and *APOB* mutation carriers was significantly different (*p* = 0.03) as well as the overall difference between the three groups (ANOVA test, *p* = 3.31 × 10^−8^). This result confirms previous findings [17]. The TC level was significantly reduced by treatment in both mutation positive and mutation negative groups by at least 35% (see Supplemental Data Table 4). The UK guidelines on management of FH dyslipidaemias recommend that the treatment goal for FH should be to reduce LDL-C by at least 50% from baseline [16], while guidelines on management of dyslipidaemias recommend that LDL-C in high risk subjects should be reduced below 2.5 mmol/l and for individuals with CVD (very high risk) below 1.8 mmol/l [25]. Data on pre- and post-treatment LDL-C was available for 104 individuals, and the 50% LDL-C reduction was achieved in 47% of the patients. Post-treatment LDL-C values were available for 176 patients of which 26 (14.8%) had LDL-C reduced below 2.5 mmol/l and 1 patient (0.6%) had LDL-C below 1.8 mmol/l (Fig. 3).

## 4 Discussion

This study has identified marked genetic heterogeneity among patients with heterozygous familial hypercholesterolaemia attending a single UK lipid clinic with a catchment population of more than 620,000 people in the Oxfordshire region. Amongst 272 unrelated patients 54 different *LDLR* mutations in 90 subjects were identified, which is comparable with the 107 different mutations identified in 232 subjects in the UK Cascade pilot project who were from five geographically dispersed sites [11]. There were 12 previously unreported mutations found in the Oxford cohort, which accounted for 22% of all *LDLR* mutations found in this study. This is also substantially higher than the 7% novel variants found in the previous UK study [11]. While this may be a result of the increased sensitivity of the current mutation detection methods, it may also reflect the genetic heterogeneity of this Oxford sample. Overall, the mutation spectrum was similar to the rest of the UK, however the most commonly occurring *LDLR* mutation in Oxford was c.301G > A (p.(Glu101Lys)), which accounted for 6% of all observed mutations, as opposed to 1% in the whole of the UK study [11]. The frequency of gross deletions/duplications within the *LDLR* was higher (10%) than previously reported (5–6%), however this was not statistically different (Fisher's exact test, *p* = 0.06). The *PCSK9* (p.(Asp374Tyr)) mutation, which is associated with a higher CHD risk than other FH-causing mutations [7], was not observed in the Oxford cohort.

A striking finding was the importance of high pre-treatment cholesterol and low triglyceride levels as predictors of the likelihood of detection of an FH-causing mutation. The detection rate of 73% in DFH compared to the 27% in PFH in patients attending the Oxford clinic was similar to that reported previously in UK patients [17,22,26,27]. Additionally, we examined the utility of the Dutch Lipid Clinic Network (DLCN) score, in identifying patients with a high or low probability of carrying an FH-causing mutation. In patients with a DLCN score indicative of Definite FH (>8), Probable FH (>5 and <8) and Possible FH (>3 and <5), the mutation detection rates were 54% vs. 39% vs. 28%, respectively, and using the AROC statistic the two approaches were not significantly different in discrimination. Combining pre-treatment TC and TG levels gave the highest likelihood of finding patients carrying FH mutations, with 100% mutation detection rate in patients with pre-treated TC above 10.0 mmol/l and with TG below 1.0 mmol/l, compared to >5% detection rate in those with pre-treated TC below 8.0 mmol/l and with TG above 2.15 mmol/l.

A proportion of the studied patients (26%) did not fulfil the Simon Broome criteria for FH as there was either no family history of hypercholesterolaemia or premature CHD or, alternatively, the family history was unknown or incomplete. There were 10 FH-causing mutations found among probands of the unclassified hypercholesterolaemic individuals (14%), and the identification of an FH-causing mutation therefore changed the diagnosis to DFH, which would have warranted cascade testing of their first-degree relatives. This finding supports the clinical utility of DNA testing, even in individuals with a low probability of being affected. We can speculate as to the likely genetic cause of the elevated cholesterol and triglyceride levels seen in the mutation negative subjects examined here. We have recently demonstrated [28], that in a significant proportion of mutation negative patients with a clinical diagnosis of FH, their elevated LDL-C can be explained by the “polygenic” contribution of 12 common LDL-C-raising variants, in genes identified through Genome Wide Association studies. Similarly it has been demonstrated that the frequencies of common triglyceride-associated variants are also significantly higher in groups of patients with different Fredrickson classification forms of hypertriglyceridaemia [29] compared with controls. Although we do not have data to address this directly, it is likely that the elevated levels of lipids seen in the Oxford no-mutation group have a polygenic and not a monogenic explanation.

A limitation of the study was that pre-treatment TC measurements were not available for 31 out of 289 participants, and pre-treatment TG levels were not recorded for 130 individuals. This was because some patients who met the criteria in other respects (e.g. personal family history of premature CHD or tendon xanthomas) were referred to the lipid clinic whilst on diet or lipid-lowering medication, and it would not have been ethical to stop medication to collect data for this study. Although both the SB and DLCN clinical diagnostic methods performed well, the clinical utility of the Simon Broome diagnostic criteria may be questioned since they require tendon xanthomata to be present for a diagnosis of DFH, and these are increasingly uncommon as patients are diagnosed at a younger age. Although the methodology used for the mutation screening is appropriate and was shown to be sensitive and robust [23], a recent report exposed some limitations to the current methods, which included false negative calling due to human error in data and sample handling [12].

The UK 2008 NICE guidelines for the management of FH recommends a target reduction in LDL-C with treatment of greater than 50% from baseline, which was achieved in less than half of patients in this study. Furthermore, reduction of LDL-C below the ESC/EAS guideline of 2.5 mmol/l for moderate risk subjects [25], was achieved in only about 15% of the patients, and only one patient had post-treatment LDL-C below the 1.8 mmol/l target for individuals at a very high risk. Inadequate LDL-C reduction in most patients highlights the need for more effective lipid-lowering drug therapy, and novel treatments, such as PCSK9 inhibition, may offer new therapeutic opportunities to achieve this [30].

## Funding

SEH holds a Chair funded by the British Heart Foundation, and SEH, and RW are supported by the BHF (PG08/008). MF is funded by an MRC CASE award with Gen-Probe Life Sciences Ltd. HAWN is a NIHR Senior Investigator. The cost of the genetic analyses was supported (in part) by the NIHR. None of the authors have any financial or personal relationships to declare that might bias this work.

## Competing interests

None.

## Figures and Tables

**Table 1:** Baseline characteristics of patients with definite FH (DFH), possible FH (PFH) and unclassified hypercholesterolaemia (UH).

Variable	DFH (*N* = 65)	*p* value (DFH vs. PFH)	PFH (*N* = 150)	*p* value (PFH vs. UH)	UH (*N* = 74)	*p* value (overall)
Gender						
% Male (N)	47.7 (34)		46.7 (70)		58.1 (43)	0.25
Mean age (SD)	58.0 (12.7)		53.8 (14.1)		54.2 (14.9)	0.12

Pre-treatment TC	9.79 (1.66)	9.79 × 10^−06^	8.71 (1.27)	0.698	8.47 (1.92)	<0.0001
Pre-treatment HDL-C	1.38 (0.35)	0.302	1.46 (0.40)	0.634	1.39 (0.39)	0.62
Pre-treatment TG	1.22 (0.56)	0.015	1.53 (0.72)	0.232	2.00 (0.81)	0.004
Pre-treatment LDL-C	6.93 (1.61)	0.005	6.12 (1.15)	0.162	5.45 (1.72)	0.0002

Post-treatment TC	5.95 (1.01)	0.008	5.46 (1.13)	0.676	5.36(1.17)	0.008
Post-treatment HDL-C	1.31 (0.36)	0.067	1.44 (0.32)	0.97	1.40 (0.46)	0.1
Post-treatment TG	1.12 (0.52)	0.206	1.25 (0.60)	0.483	1.21 (0.38)	0.42
Post-treatment LDL-C	4.1 (1.04)	9.20 × 10^−05^	3.29 (1.00)	0.537	3.65 (1.57)	0.0001

Detected mutations (in probands only)						
*LDLR* (%)	43(71.7)		30(21.1)		9(12.9)	<0.0001
*APOB* (%)	1(1.7)	1.94 × 10^−09^	8(5.6)	0.062	1(1.4)	
None (%)	16(26.7)		104(73.2)		60(85.7)	

Cholesterol concentrations were not normally distributed, and are presented as geometric means with an approximate standard deviation in brackets.

**Table 2:** Novel ***LDLR*** variants identified in the Oxford Lipid Clinic patients and the ***in silico*** prediction of their effect.

Mutation type/Exon	Variant position	PolyPhen2	SIFT	Mutation taster	Conclusion
Promoter					
	c.-121T > C	N/A	N/A	D	Transcription factor binding site disruption (publication in preparation)
Missense					
4	c.361T > A (p.(Cys121Ser))	D	D	D	FH-causing
4	c.629T > A (p.(Ile210Asn))	D	D	D	FH-causing
6	c.859G > A (p.(Gly287Ser))	D	D	D	FH-causing
9	c.1230G > T (p.(Arg410Ser))	D	D	D	FH-causing
14	c.2098G > A (p.(Asp700Asn))	P	D	D	FH-causing
17	c.2476C > A (p.(Pro826Thr))	D	D	D	FH-causing
Nonsense					
6	c.898A > T (p.(Arg300*))	N/A	N/A	D	Formation of premature stop codon
Small rearrangements					
4	c.667_693del (p.(Lys223_Cys231del))	N/A	N/A	N/A	Deletion of 9 highly conserved residues (Supplemental Data Fig. 1)
10	c.1379_1402delinsCAGCTTGACCCGC (p.(His460Profs*3))	N/A	N/A	N/A	Frame shift → premature stop codon
15	c.2187_2197del (p.(Leu729Leufs*39))	N/A	N/A	D	Frame shift → premature stop codon
Large rearrangements					
11	c.1587-?_1845+?dup	N/A	N/A	N/A	Frame shift → premature stop codon

D–probably damaging (PolyPhen2), not tolerated (SIFT), disease causing (Mutation Taster) P–possibly damaging. N/A–not applicable.

**Table 3:** FH mutation detection rate by quartile of pre-treatment TC and pre-treatment TG.

Oxford FH study					
Quartile of pre-treatment TC (mmol/l)	*N*	Mutation + ve (%)	Quartile of pre-treatment TG (mmol/l)	*N*	Mutation + ve (%)
Q1 ≤8.0	40	10 (25)	Q1 ≤1.0	42	25 (60)
Q2 8.1–8.7	41	12 (29)	Q2 1.10–1.32	38	15 (40)
Q3 8.8–9.9	44	15 (34)	Q3 1.33–2.15	39	14 (36)
Q4 >10.0	34	25 (74)	Q4 2.16–4.30	40	8 (20)
*P* value (trend)		*p* = 9.83 × 10^−5^			0.000458

Replication SBBHF study					
Quartile of pre-treatment TC (mmol/l)	*N*	Mutation + ve (%)	Quartile of post-treatment TG (mmol/l)^a^	*N*	Mutation + ve (%)
Q1 ≤8.7	76	52 (68.4)	Q1 <1	105	92 (87.6)
Q2 8.8–10.1	82	66 (80.5)	Q2 1–1.3	123	109 (88.6)
Q3 10.2–11.6	69	62 (89.9)	Q3 1.4–1.8	90	70 (77.8)
Q4 >11.6	72	65 (90.3)	Q4 >1.8	91	57 (62.6)
*P* value (trend)		0.0001			*P* = 1.80 × 10^−6^

a Pre-treatment TG were not available for SBBHF study.

**Table 4:** The FH mutation detection rate in patients diagnosed using the DLCN score, with the percentage of DFH diagnosed by the Simon Broome FH criteria in each score group.

DLCN score	Patient count	Mutation + ve (%)	DFH (%)
<3	13	3 (23)	2 (15)
3–5	69	19 (28)	9 (13)
6–8	49	19 (39)	8 (16)
>8	89	48 (54)	45 (51)
*P* value for the trend (Fisher's exact)		0.004	2.6 × 10^−7^

## Appendix A Supplementary data

Supplemental Fig. 1. 3D structure of LDL-R extracellular domains (Protein Database 1N7D, http://www.ebi.ac.uk/pdbsum/1N7D) viewed using Jmol, overlaid with conservation scores. Red indicates high conservation, purple moderate conservation, blue poor conservation. Calcium cations are shown as black dots. Residues deleted in p.(Lys223_Cys231) are shown by yellow dotted line, disulphide bridge broken by the deletion shown as green line.

Supplemental Fig. 2. Pre-treatment LDL cholesterol in patients with mutation in *LDLR* or *APOB* genes, or with no mutation identified. The difference between the groups was significant (ANOVA p = 7.76 × 10^−05^).

## Footnotes

1 Human Genes: LDLR–low density lipoprotein receptor, APOB–apolipoprotein B (including Ag(x) antigen), PCSK9–proprotein convertase subtilisin/kexin type 9.
