# Supplementary material for: Analysis of the frequency and spectrum of mutations recognised to cause familial hypercholesterolaemia in routine clinical practice in a UK specialist hospital lipid clinic
Source: Atherosclerosis. 2013 Jul;229(1):161–8. doi: 10.1016/j.atherosclerosis.2013.04.011 (PMC3701838; doi:10.1016/j.atherosclerosis.2013.04.011)
Supplement: Supplementary file 1 [file mmc1.docx]

**Supplemental Table 1.** **Baseline characteristics of patients in the SBBHF study. Cholesterol concentrations were not normally distributed, and are presented as geometric means with an approximate standard deviation in brackets.**

|  | Mutation -ve  N=81 | Mutation +ve  N=328 | Total  N=409 | P value |
| --- | --- | --- | --- | --- |
| % male  Age  Pre-treatment cholesterol  Post treatment  Cholesterol  LDL  HDL  Triglycerides | 50.6% (41)  52.4 (13.6)  9.22 (1.71)  6.39 (1.41)  4.06 (1.27)  1.37 (0.33)  1.62 (0.76) | 51.5% (169)  48.3 (13.5)  10.35 (1.88)  6.74 (1.29)  4.67 (1.24)  1.34 (0.37)  1.25 (0.55) | 51.3% (210)  49.1 (13.6)  10.13 (1.90)  6.67 (1.32)  4.54 (1.28)  1.34 (0.36)  1.32 (0.60) | 0.88  0.01  0.00003  0.03  0.00004  0.49  3.136x10^-6^ |

**Supplemental Table 2.**

**All variants found in the Oxford Lipid Clinic patients’ cohort. Variants reported for the first time are presented in bold.**

| **Gene** | **Base pair change** | **Predicted amino acid change** | **Number of carriers (number of probands)** |
| --- | --- | --- | --- |
| *APOB* | c.10580G>A | p.(Arg3527Gln) | 11(10) |
| *LDLR* | c.301G>A | p.(Glu101Lys) | 6(6) |
| *LDLR* | c.313+1G>A | N/A | 4(4) |
| *LDLR* | c.681C>G | p. (Asp227Glu) | 4(3) |
| *LDLR* | c.1444G>A | p.(Asp482Asn) | 4(3) |
| *LDLR* | c.259T>G | p.(Trp87Gly) | 3(3) |
| *LDLR* | c.680_681delAC | p.(Asp227Glyfs*12) | 3(3) |
| *LDLR* | c.1116_1119dupGGGT | p.(Gly374fs*8) | 3(3) |
| *LDLR* | c.2054C>T | p.(Pro685Leu) | 3(3) |
| *LDLR* | c.191-?_1186+?del | N/A | 3(2) |
| ***LDLR*** | **c.2187_2197del** | **p.(Leu729Leufs*39)** | **3(2)** |
| *LDLR* | c.2089G>C | p.(Ala697Pro) | 3(1) |
| *LDLR* | c.662A>G | p.(Asp221Gly) | 2(2) |
| *LDLR* | c.682G>T | p.(Glu228*) | 2(2) |
| *LDLR* | c.695-?_817+?del | N/A | 2(2) |
| *LDLR* | c.912C>G | p.(Asp304Glu) | 2(2) |
| *LDLR* | c.1846-?_2140+?del | N/A | 2(2) |
| ***LDLR*** | **c.667_693del** | **p.(Lys223_Cys231del)** | **2(1)** |
| ***LDLR*** | **c.-121T>C** | **N/A** | **1(1)** |
| *LDLR* | c.68-?_1186+?dup | N/A | 1(1) |
| *LDLR* | c.118delA | p.(Ile40Serfs*166) | 1(1) |
| *LDLR* | c.139G>A | p.(Asp47Asn) | 1(1) |
| *LDLR* | c.191-?_1186+?dup | N/A | 1(1) |
| *LDLR* | c.191-?_313+?del | N/A | 1(1) |
| ***LDLR*** | **c.361T>A** | **p.(Cys121Ser)** | **1(1)** |
| *LDLR* | c.502G>A | p.(Asp168Asn) | 1(1) |
| *LDLR* | c.621C>T | p.(Gly207Gly) | 1(1) |
| ***LDLR*** | **c.629T>A** | **p.(Ile210Asn)** | **1(1)** |
| *LDLR* | c.654_656delTGG | p.(Gly219del) | 1(1) |
| ***LDLR*** | **c.859G>A** | **p.(Gly287Ser)** | **1(1)** |
| *LDLR* | c.889A>C | p.(Asn297His) | 1(1) |
| ***LDLR*** | **c.898A>T** | **p.(Arg300*)** | **1(1)** |
| *LDLR* | c.933delA | p.(Lys311fs*59) | 1(1) |
| *LDLR* | c.1048C>T | p.(Arg350*) | 1(1) |
| *LDLR* | c.1049G>C | p.(Arg350Pro) | 1(1) |
| *LDLR* | c.1135T>C | p.(Cys379Arg) | 1(1) |
| *LDLR* | c.1215C>G | p.(Asn405Lys) | 1(1) |
| ***LDLR*** | **c.1230G>T** | **p.(Arg410Ser)** | **1(1)** |
| *LDLR* | c.1246C>T | p.(Arg416Trp) | 1(1) |
| *LDLR* | c.1285G>A | p.(Val429Met) | 1(1) |
| *LDLR* | c.1369_1372dupGACA | p.(Arg458fs*10) | 1(1) |
| ***LDLR*** | **c.1379_1402delinsCAGCTTGACCCGC** | **p.(His460Profs*3)** | **1(1)** |
| *LDLR* | c.1436T>C | p.(Leu479Pro) | 1(1) |
| *LDLR* | c.1466A>G | p.(Tyr489Cys) | 1(1) |
| *LDLR* | c.1567G>A | p.(Val523Met) | 1(1) |
| *LDLR* | c.1574A>T | p.(Asp525Val) | 1(1) |
| *LDLR* | c.1587-?_1845+?del | N/A | 1(1) |
| ***LDLR*** | **c.1587-?_1845+?dup** | **N/A** | **1(1)** |
| *LDLR* | c.1646G>A | p.(Gly549Asp) | 1(1) |
| *LDLR* | c.1694G>C | p.(Gly565Ala) | 1(1) |
| *LDLR* | c.1897C>T | p.(Arg633Cys) | 1(1) |
| *LDLR* | c.1941_1944delins1922_1934{1922_1934dup} | N/A | 1(1) |
| ***LDLR*** | **c.2098G>A** | **p.(Asp700Asn)** | **1(1)** |
| *LDLR* | c.2393_2401del | p.(Leu799_Phe801del) | 1(1) |
| ***LDLR*** | **c.2476C>A** | **p.(Pro826Thr)** | **1(1)** |
|  |  |  |  |

**Supplemental Table 3. Total cholesterol reduction after treatment in patients with or without an identified FH mutation, in DFH, PFH and UH.**

|  | **DFH** | **PFH** | **UH** |
| --- | --- | --- | --- |
| Mutation -ve | -37.5%  (-47.0 to -26.3) | -38.1%  (-41.3 to -34.7) | -35.2%  (-39.5 to -30.6) |
| Mutation +ve | -39.9%  (-44.0 to -35.5) | -41.8%  (-45.3 to -38.0) | -41.0%  (-52.2 to -27.3) |
| All | -39.3%  (-43.1 to -35.3) | -39.1%  (-41.6 to -36.4%) | -36.5%  (-40.5 to -32.2) |

All reductions statistically significant at p < 0.001

**Supplemental Figure 1. 3D structure of LDL-R extracellular domains (Protein Database 1N7D,** [**http://www.ebi.ac.uk/pdbsum/1N7D**](http://www.ebi.ac.uk/pdbsum/1N7D)**) viewed using Jmol, overlaid with conservation scores. Red indicates high conservation, purple moderate conservation, blue poor conservation. Calcium cations are shown as black dots. Residues deleted in p.(Lys223_Cys231) are shown by yellow dotted line, disulphide bridge broken by the deletion shown as green line.**

**Supplemental Figure 2. Receiver Operating Characteristic (ROC) curve comparing the accuracy of the Simon Broome FH criteria (SB) vs. the Dutch Lipid Clinic Network criteria (Dutch score) in Oxford Lipid Clinic patients and the SBBHF study combined. Chi square test p value of the area under the curve was not significant (p > 0.68).**

**Supplemental Figure 3. Pre-treatment LDL cholesterol in patients with mutation in *LDLR* or *APOB* genes, or with no mutation identified. The difference between the groups was significant (ANOVA p = 7.76 x 10^-05^).**
