# Supplementary figures and images for: Analysis of the frequency and spectrum of mutations recognised to cause familial hypercholesterolaemia in routine clinical practice in a UK specialist hospital lipid clinic
Source: Atherosclerosis. 2013 Jul;229(1):161–8. doi: 10.1016/j.atherosclerosis.2013.04.011 (PMC3701838; doi:10.1016/j.atherosclerosis.2013.04.011)

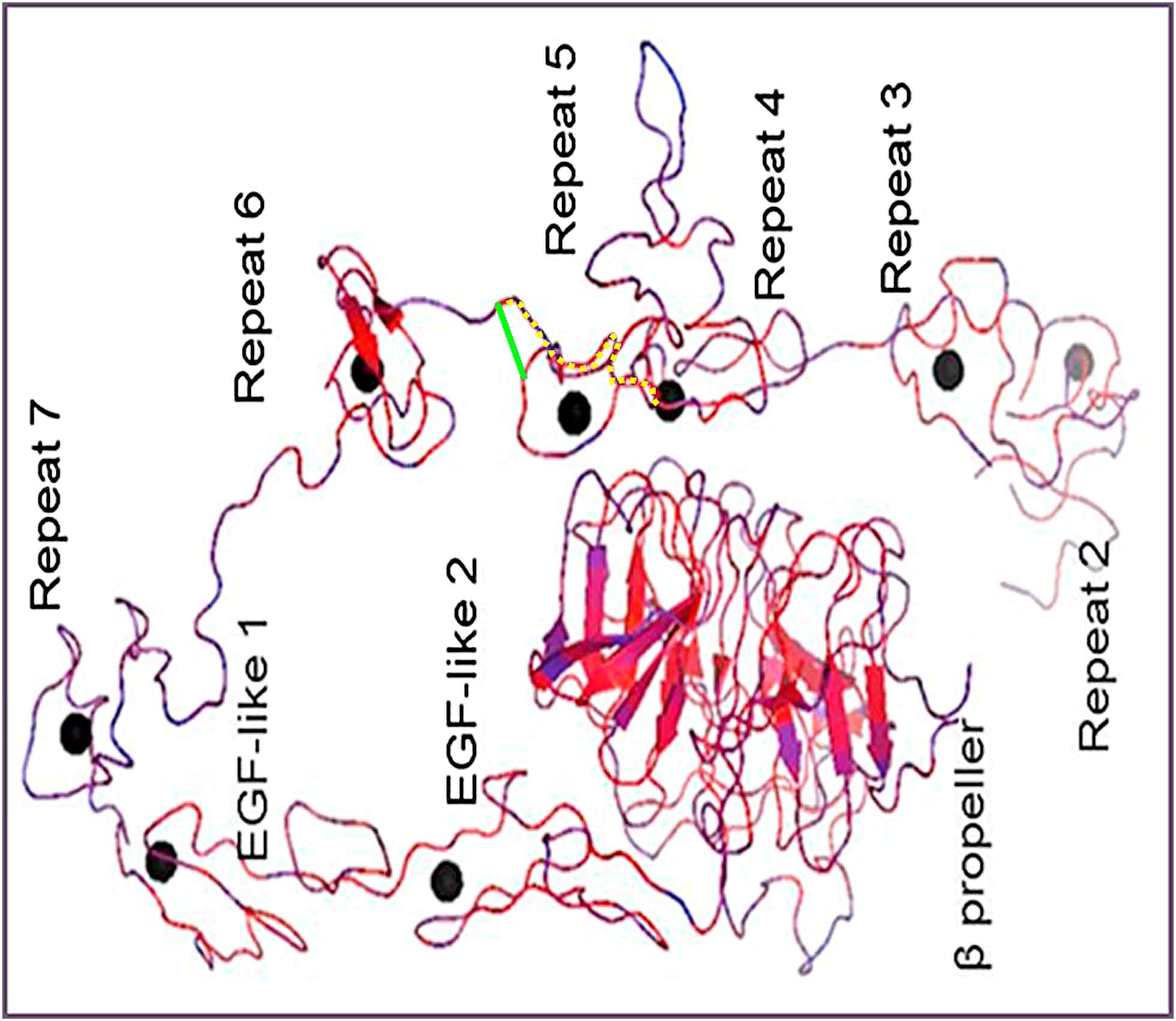

Supplement: Supplementary file 2 — Supplemental Fig. 1. 3D structure of LDL-R extracellular domains (Protein Database 1N7D, http://www.ebi.ac.uk/pdbsum/1N7D) viewed using Jmol, overlaid with conservation scores. Red indicates high conservation, purple moderate conservation, blue poor conservation. Calcium cations are shown as black dots. Residues deleted in p.(Lys223_Cys231) are shown by yellow dotted line, disulphide bridge broken by the deletion shown as green line. [file figs1.jpg]

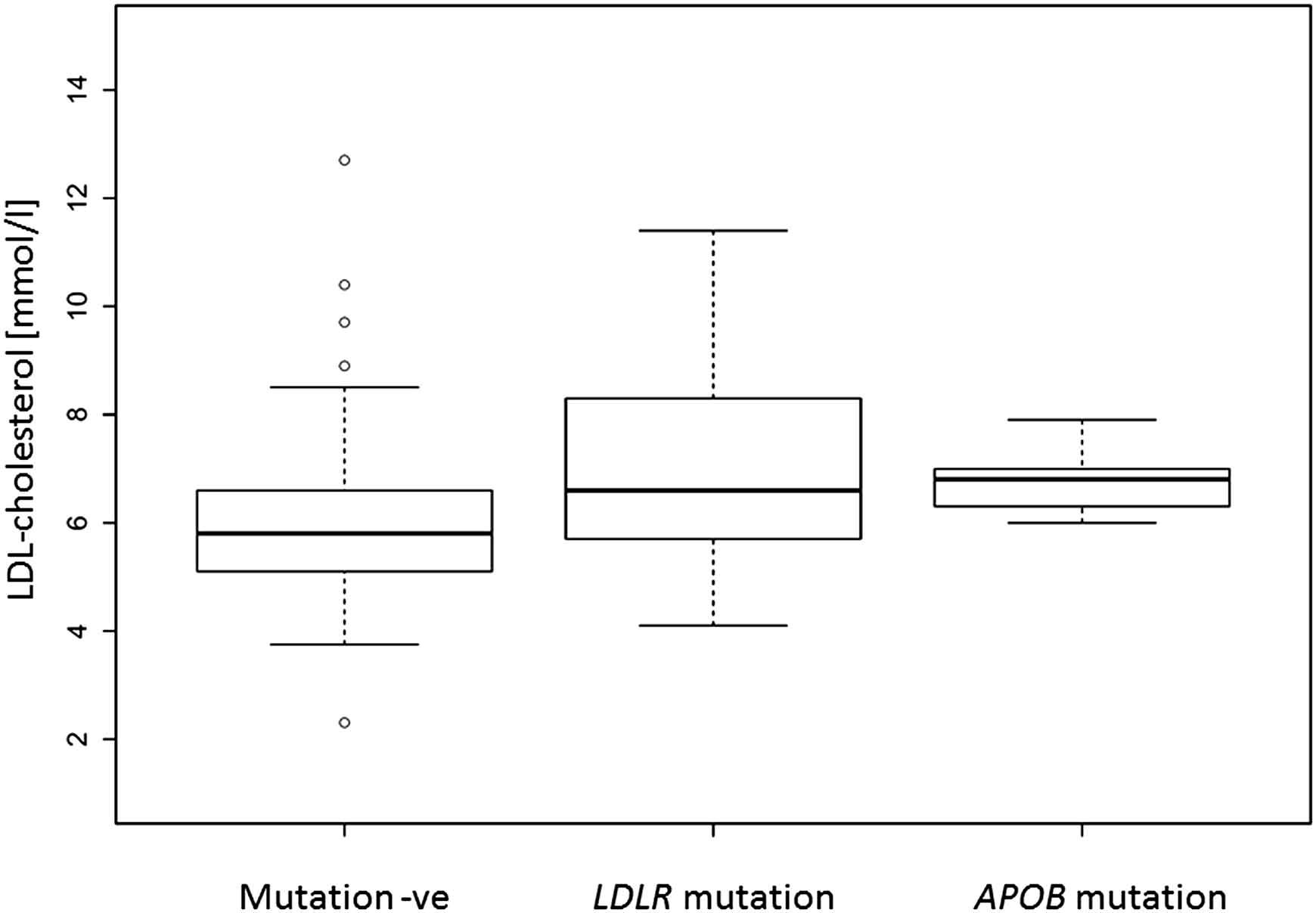

Supplement: Supplementary file 3 — Supplemental Fig. 2. Pre-treatment LDL cholesterol in patients with mutation in LDLR or APOB genes, or with no mutation identified. The difference between the groups was significant (ANOVA p = 7.76 × 10^−05^). [file figs2.jpg]
